# Significant Relationship Between Worsening Sleep and Declining Brain Metabolism in APOE4+ Individuals

**DOI:** 10.1002/alz.095707

**Published:** 2025-01-09

**Authors:** Anne E. Hogue, Daniel H. Silverman

**Affiliations:** ^1^ UCLA, Los Angeles, CA USA; ^2^ University of California, Los Angeles, Los Angeles, CA USA

## Abstract

**Background:**

We recently reported that subjects who had worsening sleep over time had a clear worsening of both memory and brain metabolism compared to subjects whose sleep improved over time, and metabolic decline was particularly significant in the posterior cingulate cortex (PCC) (Alzheimer’s Dement. 2023; 19(Suppl. 24):e082867), an area of the brain that metabolically declines in the earliest stages of Alzheimer’s disease. Here we investigate the impact of worsening sleep on regional metabolic decline in Apolipoprotein E epsilon 4 positive (APOE4+) subjects compared to APOE4‐ subjects.

**Method:**

A consecutive series of 2,005 cognitively normal and mildly impaired subjects from more than 50 North American sites participating in the first three phases of the Alzheimer’s Disease Neuroimaging Initiative were studied. Changes in cognition assessed through formal neuropsychological testing, and in regional cerebral metabolism assessed through brain PET using the radiotracer [F‐18]fluorodeoxyglucose were examined. Activities in 47 standardized volumes of interest (sVOI’s), as normalized to each subject’s pons activity, were longitudinally measured.

**Result:**

In this consecutive series of subjects, 211 were found to have had a change in insomnia status during their participation: 83 who were documented to have insomnia at baseline that was subsequently alleviated (“improving sleep”), and 128 who were documented to have no insomnia at baseline but developed it during the study period (“worsening sleep”); of the 211, 62 (29%) underwent FDG‐PET scans during both baseline insomnia and subsequent insomnia‐free status, or both baseline normal sleep and subsequent insomnia status periods. Within this group, 27 subjects were APOE4+ and 35 were APOE‐, with 59% of the APOE4+ subjects belonging to the worsening sleep group, and 41% within the improving sleep group. In the worsening sleep group with APOE4+ status, there was metabolic decline in bilateral mesial temporal and right thalamic regions (p&lt;0.0001), but not among those with APOE4‐ status (p&gt;0.50).

**Conclusion:**

Worsening sleep was associated with regionally specific cerebral metabolic decline, as described above, in APOE4+ but not APOE4‐ non‐demented subjects, suggesting that poor sleep status may pose especially heightened risk of decline for those bearing APOE4 alleles.

